# A novel antagonist of p75NTR reduces peripheral expansion and CNS trafficking of pro-inflammatory monocytes and spares function after traumatic brain injury

**DOI:** 10.1186/s12974-016-0544-4

**Published:** 2016-04-22

**Authors:** Sangmi Lee, Aaron Mattingly, Amity Lin, Jeffrey Sacramento, Leda Mannent, Marie-Noelle Castel, Benoit Canolle, Sandrine Delbary-Gossart, Badia Ferzaz, Josh M. Morganti, Susanna Rosi, Adam R. Ferguson, Geoffrey T. Manley, Jacqueline C. Bresnahan, Michael S. Beattie

**Affiliations:** Department of Neurological Surgery, Brain and Spinal Injury Center, University of California at San Francisco, Box 0899, 1001 Potrero Ave, Bldg 1, Rm 101, San Francisco, CA 94143-0899 USA; Biomedical Science Graduate Program, University of California at San Francisco, San Francisco, CA 94143-0899 USA; Early to Candidate, Sanofi Research, 195 route d’Espagne, Chilly-Mazarin, France; Evotec, 195 route d’Espagne, 31036 Toulouse cedex, France

**Keywords:** Traumatic brain injury (TBI), p75 neurotrophin receptor (p75NTR), Inflammatory responses, Pro-inflammatory monocytes, Neuroprotection, Therapeutic target

## Abstract

**Background:**

Traumatic brain injury (TBI) results in long-term neurological deficits, which may be mediated in part by pro-inflammatory responses in both the injured brain and the circulation. Inflammation may be involved in the subsequent development of neurodegenerative diseases and post-injury seizures. The p75 neurotrophin receptor (p75NTR) has multiple biological functions, affecting cell survival, apoptotic cell death, axonal growth, and degeneration in pathological conditions. We recently found that EVT901, a novel piperazine derivative that inhibits p75NTR oligomerization, is neuroprotective, reduces microglial activation, and improves outcomes in two models of TBI in rats. Since TBI elicits both CNS and peripheral inflammation, we used a mouse model of TBI to examine whether EVT901 would affect peripheral immune responses and trafficking to the injured brain**.**

**Methods:**

Cortical contusion injury (CCI)-TBI of the sensory/motor cortex was induced in C57Bl/6 wild-type mice and CCR2^+/RFP^ heterozygote transgenic mice, followed by treatment with EVT901, a selective antagonist of p75NTR, or vehicle by i.p. injection at 4 h after injury and then daily for 7 days. Brain and blood were collected at 1 and 6 weeks after injury. Flow cytometry and histological analysis were used to determine peripheral immune responses and trafficking of peripheral immune cells into the lesion site at 1 and 6 weeks after TBI. A battery of behavioral tests administered over 6 weeks was used to evaluate neurological outcome, and stereological estimation of brain tissue volume at 6 weeks was used to assess tissue damage. Finally, multivariate principal components analysis (PCA) was used to evaluate the relationships between inflammatory events, EVT901 treatment, and neurological outcomes.

**Results:**

EVT901 is neuroprotective in mouse CCI-TBI and dramatically reduced the early trafficking of CCR2+ and pro-inflammatory monocytes into the lesion site. EVT901 reduced the number of CD45^high^CD11b+ and CD45^high^F4/80+ cells in the injured brain at 6 weeks. TBI produced a significant increase in peripheral pro-inflammatory monocytes (Ly6C^int-high^ pro-inflammatory monocytes), and this peripheral effect was also blocked by EVT901 treatment. Further, we found that blocking p75NTR with EVT901 reduces the expansion of pro-inflammatory monocytes, and their response to LPS in vitro, supporting the idea that there is a peripheral EVT901 effect that blunts inflammation. Further, 1 week of EVT901 blocks the expansion of pro-inflammatory monocytes in the circulation after TBI, reduces the number of multiple subsets of pro-inflammatory monocytes that enter the injury site at 1 and 6 weeks post-injury, and is neuroprotective, as it was in the rat.

**Conclusions:**

Together, these findings suggest that p75NTR signaling participates in the production of the peripheral pro-inflammatory response to CNS injury and implicates p75NTR as a part of the pro-inflammatory cascade. Thus, the neuroprotective effects of p75NTR antagonists might be due to a combination of central and peripheral effects, and p75NTR may play a role in the production of peripheral inflammation in addition to its many other biological roles. Thus, p75NTR may be a therapeutic target in human TBI.

**Electronic supplementary material:**

The online version of this article (doi:10.1186/s12974-016-0544-4) contains supplementary material, which is available to authorized users.

## Background

Traumatic brain injury (TBI) is a huge public health problem; 1.7 million people sustain a brain injury annually, and TBI is a contributing factor to a third of all injury-related deaths (CDC statistics). Mild to severe TBI patients often suffer long-term disabilities, including cognitive, sensory and motor dysfunction, dementia, fatigue, epilepsy, headaches, depression, and regression of social behavior [1–6]. Despite the large numbers of TBI patients, there is no effective pharmacological treatment available. Understanding the pathogenesis of TBI is critical; TBI triggers complex and heterogenous pathologic events initiated by the mechanical injury and hemorrhage and the subsequent progressive secondary injury processes [3]. In addition to necrotic and apoptotic cell death cascades [7, 8], these include robust inflammatory responses in the injured brain including activation of resident microglia and subsequent infiltration of peripheral leukocytes. Chronic TBI patients often display long-lasting inflammatory responses continuing for months to years [9, 10]. Such inflammation may contribute to structural brain abnormalities, cognitive dysfunction [10], and white matter degeneration (e.g., corpus callosum shrinkage) [11]. Thus, neuroinflammation likely plays a critical role in the pathology of chronic TBI.

TBI also increases pro-inflammatory responses in the peripheral circulation. A recent clinical study shows that trauma increased the level of MCP-1 (monocyte chemotactic protein-1; also known as CCL2) in the peripheral blood suggesting that peripheral immune system activation may provide a surrogate biological index for TBI impairment [12]. Traumatic spinal cord injury (SCI) increases the number of myeloid cells in the circulation, a pressage to the infiltration of myeloid cells into the injured spinal cord [13]. Ischemic brain injury releases soluble damage-associated molecular pattern (DAMP) molecules that induce peripheral inflammation via toll-like receptors (TLRs) on peripheral leukocytes [13, 14]. These data suggest that monitoring peripheral myeloid cells may predict not only the pro-inflammatory responses of peripheral blood cells but also the trafficking of myeloid cells into the CNS. Indeed, some studies report that peripheral blood cells increase pro-inflammatory genes in response to brain injury [15–17]. Furthermore, peripheral blood mononuclear cells (PBMCs) isolated from post-traumatic stress disorder (PTSD) patients have elevated spontaneous pro-inflammatory cytokine expression, e.g., interleukin-1 beta (IL-1β), interleukin-6 (IL-6), and tumor necrosis factor-alpha (TNF-α), providing evidence that PBMCs can be a circulating biological indicator or biomarker of neuroinflammation after injury [18]. Interestingly, several preclinical studies of SCI and TBI show that attenuating leukocyte infiltration into the injured CNS in the acute period after trauma often results in not only inhibition of inflammatory responses in the injured tissue but also better neurological outcomes in the long term [13, 19–24]. Thus, peripheral inflammation initiated in response to CNS trauma can feed forward and exacerbate dysfunction. Further, chronic peripheral inflammation can lead to immune suppression, which is a common feature of brain and spinal cord injuries, adding significantly to post-injury morbidities [25].

We and others have previously targeted secondary injury processes associated with activation of the p75 neurotrophin receptor (p75NTR) as a neuroprotective strategy after brain and spinal cord injury [26–29]. p75NTR, a low-affinity neurotrophin receptor, is a transmembrane protein in the TNF receptor superfamily (TNFRS-16) [30, 31]. p75NTR binds to all mature neurotrophins (NTs) with a low affinity; however, the dimerization with its multiple co-receptors conveys high-affinity for different ligands and provides ligand selectivity [32]. For example, p75NTR-Trks (A, B, and C) dimers have much greater affinity for mature NTs, mediating cell survival and neurite outgrowth [32–34]. However, when p75NTR forms complexes with sortilin, it has a high affinity for the pro-neurotrophins (pro-nerve growth factor (NGF) and pro-brain-derived neurotrophic factor (BDNF)) and mediates apoptotic cell death [32, 35], while p75NTR-Nogo/Lingo 1 complexes prefer to bind with myelin-derived ligands (oligodendrocyte myelin glycoprotein (OMgp) and myelin-associated glycoprotein (MAG)), resulting in the inhibition of axonal regeneration [36, 37].

p75NTR is widely expressed in the developing brain, but its expression is restricted to few regions in the adult brain [38–40]. However, it is up-regulated, along with its ligand pro-NGF [26, 41] in various CNS pathologies and after injury [32]. p75NTR null mice show reduced cortical neuronal cell death after close axotomy [27], reduced tissue damage and motor deficits after TBI [42], and reduced oligodendrocyte apoptosis after SCI [26], and treatment with a p75NTR antagonist results in oligodendrocyte protection and improvement in functional recovery after SCI [29]. A recent study has also shown protection and improved recovery in p75NTR^−/−^ mice [42]. We have recently studied the effects of a novel p75NTR antagonist, EVT901, on two models of TBI in rats [43]. EVT901 is a novel piperazine-derived compound, which interacts with the first cysteine-rich domain of the extracellular region, interfering with p75NTR oligomerization. In vitro binding studies showed that EVT901 targets p75NTR, but not other members of the TNFR superfamily tested (TNFR1, TNFR2, HVEM, 4-1BB, LTR, DR5, BCMA, Fas) [43]. Further, treatment of both controlled cortical impact (CCI) and lateral fluid percussion (LFP) TBI in rats resulted in a dramatic preservation of brain tissue volume, inhibition of cell death in both oligodendrocytes and cortical neurons, and promotion of neurological recovery [43]. Although p75NTR has rarely been observed on microglia, treatment with EVT901 dramatically reduced microglial activation in the lesion area, suggesting that p75NTR blockade could reduce central neuroinflammation. There is evidence for expression of p75NTR in leukocytes [44–46] and mesenchymal stem cells [47, 48], and in light of the evidence linking CNS and peripheral inflammation, we wondered whether the dramatic effects of EVT901 might reflect an additional action on peripheral immune responses to TBI. We chose a CCI-TBI since cortical contusions are common in human TBI, and this type of injury is characterized by a “blooming” of blood-brain barrier breakdown and hemorrhage, with many peripheral cells entering the brain over the first few days after injury [19, 49]. We found that mouse monocytes express p75NTR and that TBI increases their number at a week following injury, while sham injuries do not. One week of treatment with EVT901 blocks this effect of TBI, suggesting a CNS injury-specific effect. We found that blocking p75NTR with EVT901 reduces the expansion of pro-inflammatory monocytes, and their response to lipopolysaccharide (LPS) in vitro, supporting the idea that there is a peripheral EVT901 effect that blunts inflammation. Further, one week of EVT901 blocks the expansion of pro-inflammatory monocytes in the circulation after TBI, reduces the number of multiple subsets of pro-inflammatory monocytes that enter the injury site at 1 and 6 weeks post-injury, and is neuroprotective, as it was in the rat. Together, these results support the idea that there is a central-to-peripheral-to-central loop of inflammatory responses that contribute to CNS dysfunction after injury and identify p75NTR as a target in both central and peripheral inflammation. Thus, p75NTR may be a unique therapeutic target for immune modulation in human TBI.

## Methods

### Controlled cortical impact TBI

Adult C57BL/6 wild-type (WT) mice (3–5 months old, Jackson Laboratory) and CCR2^+/RFP^ transgenic heterozygous mice (backcrossed with C57Bl/6 mice; [50]) were used for this study. Mice were housed individually and maintained on a 12-h light/dark cycle with food and water. All animal experiments were approved by the Institutional Laboratory Animal Care and Use Committee of University of California San Francisco and performed in compliance with NIH guidelines. Surgical procedures were carried out aseptically under isoflurane anesthesia. The toe pinch-reflex test was used to determine the effectiveness of the anesthetic prior to surgery. Lacrilube ophthalmic ointment (Allergan Pharmaceuticals, Irvine, CA) was applied to the eyes prior to surgery. Body temperature was monitored using a rectal thermal probe and maintained at 37.5 ± 0.5 °C using a heating pad.

To generate the TBI, a controlled cortical impact (CCI) device (Custom Design & Fabrication, Inc., Sandston, VA) was used as described previously [43, 51]. Briefly, mice were mounted in a Kopf stereotaxic frame under isoflurane anesthesia (5 % induction, 1.5–2 % maintenance). A unilateral craniectomy (4.0-mm diameter) was produced in the skull between bregma (+2 to −2 mm) and between 1.0 and 3.0 mm laterally from bregma using a drill. Moderate contusion brain injury was generated using the CCI device oriented at a 60° angle to the cortical surface and with a 3-mm-diameter impactor tip, over the left somato-motor cortex. A 2.0-mm displacement at 4 m/s velocity with a dwell time of 150 ms was used. The injury sites were closed, and the animals maintained at 37.5 °C in a Thermocare®, Intensive Care Unit with Dome Cover (Thermocare, Inclined Village, NV). Sham-injury controls had all procedures, including the craniectomy. Animals were administered 50 mg/kg of cefazolin (Ancef, Novation, LCC, Irving, TX) perioperatively and 1 day post-operatively and 1.2 mg/kg of buprenorphine HCl CIII SR (Zoopharm, Windsor, CO, USA) once perioperatively.

### Drug treatment

One milligram per kilogram of EVT901 (Evotec, France) or vehicle (PBS) was injected i.p. once per day for 7 days starting at 4 h post-injury. This dose was chosen based on our previous study [43].

### Measurement of RFP+ monocyte signals from CCR2^+/RFP^ Tg mice

CCR2+/RFP Tg mice were euthanized at 7 days after TBI and transcardially perfused with PBS, followed by 4 % paraformaldehyde. Brains were removed, post-fixed, and cryoprotected in 30 % sucrose. Thirty-micrometer sections were cut on a cryostat (Microm HM550, Thermo Fisher Scientific, Cambridge, MA, USA). To quantitate the infiltrating RFP+ monocytes, a total of five brain sections from every 10th section from the region of interest (ROI; bregma 1.5 to −1.5 mm) were used. After washing with PBS, brain tissue sections were mounted with mounting media (Prolong Gold with DAPI, Invitrogen, Grand Island, NY, USA). Brain tissue sections were photographed with ×20 objective on a BIOREVO all-in-one fluorescence microscope with a BZ-9000 Generation II analyzer (Keyence microscopes, Itasca, IL, USA), and the stitched image from a whole brain section was obtained using the Image Stitching Function, which links *XY* coordinate positions and is able to create a wide-view image. To measure the infiltrating RFP+ monocytes in the injured brain in CCR2^+/RFP^ Tg mice, we used proportional area (P.A.) measurements based on previous studies with minor modifications [21, 52]. Briefly, a stitched image was opened using the BZ-9000 Generation II analyzer (BZ-9000 Generation II, Keyence microscope). The “hybrid cell count” function for fluorescence image types was used. To determine the specific target area, the ipsilateral or contralateral hemisphere was contoured using the “target area” function. After setting the threshold on the RFP-positive signal on the image, the RFP-positive area and the thresholded area were derived. The total areas from the ipsilateral and contralateral hemispheres were measured by the “area measurement” function. P.A. was then calculated as a ratio: (the area of the RFP signal/total area of the ipsilateral or contralateral tissue)*100. A total of 16 CCR^+/RFP^ Tg mice were used (*n* = 5 per TBI groups, vehicle vs EVT901; and *n* = 3 per sham groups, vehicle vs EVT901).

### Histology and immunocytochemistry for the CCI-TBI subjects

C57Bl/6 mice were euthanized at 7 days post-injury and transcardially perfused with PBS, followed by 4 % paraformaldehyde. Brains were removed, post-fixed, and cryoprotected in 30 % sucrose. Thirty-micrometer sections were cut on a cryostat (Microm HM550, Thermo Fisher Scientific). Brain sections were treated with blocking buffer (10 % goat serum/0.1 % BSA/0.01 % Triton X-100) for 1 h at RT and stained O/N at 4 °C with antibodies against CD45-FITC (1:200, eBioscience, San Diego, CA, USA), CCR2 (1:100, Abcam, Cambridge, MA, USA), CD11b (1:200, Millipore, Darmstadt, Germany), and F4/80 (1:200, AbD Serotec, Raleigh, NC). After washing with PBS, three times, secondary Abs (1:200; anti-Rabbit-Cys3; Jackson ImmunoLaboratories, West Grove, PA, USA, anti-Hamster rat-Cys3; Jackson ImmunoLaboratories) were added and incubated for 1 h at RT. Slides were washed with PBS for 5 min, three times, and then coverslipped with mounting media with DAPI (Prolong Gold with DAPI, Invitrogen). The stained brain tissue sections were photographed with a ×20 objective using the BIOREVO all-in-one fluorescence microscope (BZ-9000 Generation II, Keyence microscope), and positive signal was measured using BZ-9000 Generation II analyzer (Keyence).

### Measurement of P.A. for double-positive signals from immuno-stained tissue sections

To quantify the double-positive cells (CD45+ with CCR2+, or F4/80+) in the injured brain tissues, we applied P.A. measurements using the “single extraction” function using the BZ-9000 Generation II analyzer (BZ-9000 Generation II, Keyence microscope). Briefly, a total of five brain tissue sections from every 10th section from the regions of interest (ROI; from bregma +1.5 to −1.5 mm) were selected for measuring the P.A. of immuno-stained brain sections from the ROI. Using the hybrid cell count function, for fluorescence images, the target area was specified, and the ipsilateral or contralateral hemisphere was contoured and the threshold set for CD45-positive signal, CCR2-positive signal, or F4/80-positive signal on the image. The total area of the ipsilateral and contralateral hemisphere was then measured. The P.A. was calculated for each hemisphere as a ratio: (the area of double-positive signal/total area of the ipsilateral tissue)*100. A total 16 C57Bl/6 WT mice were used: *n* = 5 per TBI group (vehicle vs EVT901) and *n* = 3 for sham groups (vehicle vs EVT901).

### Determination of brain tissue damage

To determine if EVT901 protects the brain from damage after TBI, we measured the areas of the injured brain at 7 days after TBI with or without EVT901. A total of five sections from every 10th brain sections through the ROI (bregma 1.5 to −1.5 mm) were selected. The 30-μm-thick brain tissue sections were stained with cresyl violet and mounted with DPX mounting media (Sigma). The total area of the ipsilateral and contralateral hemispheres was measured using the “area measurement” function using BZ-9000 Generation II analyzer (BZ-9000 Generation II, Keyence microscope). Brain damage was determined as (area of the ipsilateral hemisphere/area of the contralateral hemisphere)*100. A total of 32 mice (16 CCR^+/RFP^ Tg mice and 16 C57Bl/6 WT mice) were used: *n* = 5 per TBI group (vehicle vs EVT901) and *n* = 3 for sham groups (vehicle vs EVT901).

### Flow cytometric analysis

#### Determination of RFP-positive cells in the circulation

To examine if TBI affected RFP-positive monocytes in the circulation, we used flow cytometry with leukocytes isolated from CCR^+/RFP^ Tg mice. Blood was obtained by cardiac puncture from CCR^+/RFP^ Tg mice at 7 days after TBI with or without EVT901 treatment. Leukocytes were isolated as described previously [13]. Briefly, 200 μl of blood was treated with 2 ml of ×1 RBC lysis buffer (eBioscience) for 5 min at RT and 20 ml of Dulbecco’s phosphate-buffered saline (DPBS) (cell culture facility at UCSF) were added. Leukocytes were obtained by centrifuging at 350×*g* for 5 min. The cell pellet was re-suspended with Fluorescence-activated cell sorter (FACS) buffer (0.02 % NaN_3_/1 % FBS/DPBS, pH 7.4). The isolated leukocytes were run with flow cytometry (LSRII, BD Bioscience, San Jose, CA, USA), and RFP+ cells were determined. For negative control, leukocytes were isolated from WT mice and run with flow cytometry. To determine cell viability, aqua amine reactive dye (AARD) (LIVE/DEAD® Fixable-Aqua Dead Cell Stain Kit, Invitrogen) was used. A total of 16 CCR^+/RFP^ Tg mice were used: *n* = 5 per TBI group (vehicle vs EVT901) and *n* = 2–3 for sham group (vehicle vs EVT901).

#### Determination of p75NTR expression in the circulation after TBI

To examine if TBI up-regulates p75NTR expression in the circulation, we used flow cytometry with leukocytes isolated from C57Bl/6 mice at 1 and 6 weeks after TBI with or without EVT901. The leukocytes were isolated as described earlier. The isolated cells were incubated with AARD according to the manufacturer’s protocol. For intracellular staining, the isolated leukocytes were fixed with IC fixation buffer (eBioscience) and treated with permeabilization buffer (eBioscience). The fixed leukocytes were stained with rabbit anti-p75NTR (Abcam) for 30 min at 4 °C and then stained with anti-rabbit-PE Ab (Invitrogen) for 30 min at 4 °C. The p75NTR-expressing cells were identified by flow cytometry (LSRII, BD Bioscience). Non-specific signal was determined using isotype control Ab. A total of 34 C57Bl/6 WT mice were used: *n* = 4–5 per TBI group (vehicle vs EVT901) and *n* = 3–5 for sham groups (vehicle vs EVT901).

#### Determination of Ly6C^high^ monocytes in the circulation

To examine if TBI affects Ly6C^high^ inflammatory monocytes in the circulation, we used flow cytometry with leukocytes isolated from C57Bl/6 mice at 1 and 6 weeks after TBI with or without EVT901 treatment. The isolated cells were incubated with AARD according to the manufacturer’s protocol. The leukocytes were stained with anti-mouse CD16/32 Fc blocking Ab (1:10, eBioscience) at 4 °C for 10 min. After adding anti-Ly6C-PE/Cy7 Ab (BioLegend) and anti-CD11b-PE/Cy5 Ab (eBioscience), the leukocytes were incubated at 4 °C for 30 min. After washing with PBS, the cells were re-suspended in fixative (1 % platelet function assay (PFA)). The stained leukocytes were identified by flow cytometry (LSRII, BD Bioscience). Non-specific signal was determined using isotype control Ab. A total of 33 C57Bl/6 WT mice were used: *n* = 4–7 per TBI group (vehicle vs EVT901) and *n* = 3 for sham groups (vehicle vs EVT901).

#### Determination of the recruitment of monocytes into the injured brain

To determine if TBI induces recruitment of Ly6C^high^ inflammatory monocytes into the injured brain, we used flow cytometric analysis. Brain cells were obtained as described previously [53, 54]. Briefly, mice were perfused with PBS, and brains were obtained from C57Bl/6 WT mice at 1 and 6 weeks after TBI with or without EVT901 treatment. The ipsilateral hemisphere was dissected in the HBSS media and mechanically dissociated with the syringe followed by pipetting with 1-ml tips. To the cell suspensions, DNase I (0.025 U/ml of final concentration) and collagenases (0.05 % of final concentration) were added and incubated for 15 min at 37 °C. The cell suspensions were centrifuged, and the cell pellets were re-suspended with 1 % FBS/HBSS. The cell suspensions were strained in a 40-μm nylon cell strainer (Becton Dickinson) and centrifuged at 1500 rpm for 5 min. The cells were re-suspended with 30 % Isotonic Percoll solution (GE Healthcare Bio-Science AB, Uppsala, Sweden) and overlay in the 70 % Percoll solution followed by centrifuging at 500×*g* for 25 min at RT without braking. The cells in the interphase were obtained and re-suspended with FACS buffer (0.02 % NaN_3_/5 % FBS in DPBS). The isolated cells were incubated with AARD according to the manufacturer’s protocol. The cells were pre-stained with anti-mouse CD16/32 Fc blocking Ab (1:10, eBioscience) at 4 °C for 10 min followed by adding Abs: anti-CD45-BV605 (BioLegend, San Diego, CA, USA), anti-Ly6C-PE/Cy7 (BioLegend), anti-CD11b-PE/Cy5 (eBioscience), and F4/80-BV421 (Biolegend). After incubating at 4 °C for 30 min, the cells were washed and re-suspended with a fixative (1 % PFA). The stained leukocytes were identified by flow cytometry (LSRII, BD Bioscience). To remove false-positive signals from multiple fluorochromes, each fluorescence compensation control was run by flow cytometry (LSRII, BD Bioscience). Non-specific signal was determined using isotype control Ab. A total of 36 C57Bl/6 WT mice were used: *n* = 5–7 per TBI group (vehicle vs EVT901) and *n* = 3–4 for sham groups (vehicle vs EVT901).

#### Data analysis

Flowjo software (Tree Star, Ashland, OR) was used for analyzing data from flow cytometry. Firstly, a fluorescence compensation was processed with Flowjo software with each fluorochrome control. For blood cells, at least 100,000 events were counted. Leukocytes were initially gated by their characteristic forward and side scatter profiles, which represent size and granularity, respectively. Viable cells were determined by AARD non-labeled cells. For brain cells, 500,000 events were counted. The brain cells were initially gated by live cells. Gated cells were then analyzed for fluorescent intensity.

To determine absolute number of total cells, the cell concentration was calculated with counting beads (CountBright™ Absolute Counting Beads for flow cytometry, Molecular Probes) according to the manufacturer’s protocol.

### EVT901 effects on p75NTR expression by LPS in vitro

To determine if p75NTR is involved in the pro-inflammatory responses of leukocytes, we used an in vitro culture system. Blood was obtained from C57Bl/6 mice. Leukocytes were isolated as described previously [13]. Briefly, blood was obtained by cardiac puncture and was treated with ×1 RBC lysis buffer (eBioscience) for 5 min at RT and added DPBS (cell culture facility in UCSF). Leukocytes were obtained by centrifuging at 350×*g* for 5 min. The cell pellet was re-suspended with RPMI media with 1 % FBS. 1 × 10^6^ cells were treated with LPS (10 ng/ml) and EVT901 (30 nM), EVT901 (30 nM) alone, or vehicle alone. After 24-h incubation at 37 °C, cells were isolated by centrifugation and used for flow cytometric analysis. The supernatant was used for TNFα ELISA.

The isolated cells were stained with CD11b-PE/Cy5 (eBioscience), a pan monocyte/macrophage marker, for 30 min at 4 °C. After washing with PBS, the leukocytes were fixed with IC fixation buffer (eBioscience). And then, the cells were placed in permeabilization buffer (eBioscience). The fixed leukocytes were stained with rabbit anti-p75NTR (Abcam) for 30 min at 4 °C and then stained with anti-rabbit-PE Ab (Invitrogen) for 30 min at 4 °C. The p75NTR expressing CD11b+ cells were identified by flow cytometry (LSRII, BD Bioscience).

The cell supernatant was used for determining TNFα production using mTNFα ELISA kit (Invitrogen) according to a manufacturer’s manual. Briefly, 50 μl of standard diluent buffer was added in the TNFα pre-coated eight-well strips followed by adding 50 μl of cell supernatant. And then, 50 μl of biotinylated mouse TNFα Biotin Conjugate solution was added and incubated for 90 min at RT. After washing with buffer, 100 μl of streptavidin-HRP working solution was added in the wells and incubated for 30 min at RT. After washing, the wells were incubated with 100 μl of Stabilized Chromogen for 30 min at RT, and then, the reaction stopped by adding 100 μl Stop solution. The wells were read on a 450-nm plate reader using Magellan software (GENios plate reader, TECAN, Switzerland). A total of six mice were used for the in vitro experiments. Data were obtained from three independent in vitro experiments.

### Volumetric analysis: the Cavalieri probe method

To determine if EVT901 affected tissue damage after TBI, the estimated volume of the injured brain was measured by systematic volumetric analysis. Briefly, at 6 weeks after TBI, the brains of PFA-perfused animals were collected and cut at 30 μm as described above. For each mouse, eight brain tissue sections (every 10th section between +1.5 and −1.5 mm from Bregma) were chosen for stereological analysis and stained with cresyl violet. The Cavalieri principle was used to generate unbiased estimates of volume using Stereo Investigator software (MBF Bioscience, Williston, VT). As a Cavalieri probe, a 150-μm square grid was placed over the brain tissue section and all the grid points that overlay the areas of interest were marked. The area of the cerebral cortex was indicated as in Fig. 8b (red for ipsilateral to the TBI, blue for contralateral). All eight brain tissue sections were evaluated by marking the grid points, which generated unbiased estimations of area. The volume was estimated by summing the areas and multiplying by the tissue thickness using Stereo investigator software (MBF Bioscience). Gunderson Coefficient of Error, *m* = 1, was evaluated to determine the accuracy of the stereological estimation and was under 0.05 (not shown) in this study (*n* = 4–5 per group).

### Behavioral outcome measures

A battery of behavioral tests was performed to assess motor function after TBI. A total of *N* = 10–12 per group were used for the behavioral testing (groups: TBI with and without EVT901 and sham with and without EVT901).

#### Paw placement test

Paw placement assessments were performed as previously described [43, 51]. Briefly, mice were placed in a clear plastic cylinder with two mirrors placed at angles such that both sides of the mice were clearly visible. Mouse behaviors were recorded with a digital camera for 3 min, and the number of times the mice placed its left, right, or both forepaws against the cylinder during weight supported movements was recorded. Individual placements were scored as either “left” or “right” when 0.5 s or more passed without the other limb contacting the cylinder. If both forepaws were used for weight-supported movements within 0.5 s of each other, a score of “both” was given. Scoring was performed using video playback by trained raters who were blind to experimental condition. Mice were tested before surgery, at 1 day after TBI and then weekly thereafter for 6 weeks.

#### IBB forelimb rating scale (cereal test)

Fine forelimb motor function was assessed using the Irvine, Beatties, and Bresnahan (IBB) cereal eating test as described previously with minor modification [51, 55, 56]. Briefly, mice were individually placed in their home cage. Consistently, sized spherical- and doughnut-shaped pieces of cereal were given to mice. Eating behavior was recorded while consuming both cereal shapes. Paw use was evaluated following the standardized scoring system for common forelimb behaviors including joint position, object support, digit movement, and grasping technique. An IBB score was assigned using the 10 point (0–9) scale as previously described [56].

#### Beam-walking test

Mice were evaluated for motor deficits after TBI using the beam-walking test, which can discriminate fine motor coordination differences between injured and sham-operated mice [57, 58]. The beam-walking device consists of a narrow plastic beam (5 mm wide and 120 cm in length) suspended 1 m above the ground over a foam pad. The mouse was placed on the end of the beam, and the number of footfaults for the forelimb contralateral to the brain injury was assessed while walking the length of the beam. A basal level of performance was achieved following 3 days of pre-training prior to surgery, with an acceptance level of fewer than three footfaults per trial. The test was performed at 1 day after TBI and then weekly for 6 weeks.

#### Inclined beam-walking test

An inclined beam-walking test as described in Chang et al. [59] was also used. Mice were placed at the high end of a 120-cm-long plastic beam (5 mm wide) set at 10° above horizontal, and the number of footfaults for the forelimb contralateral to the TBI was assessed while walking the length of the beam.

#### Rearing test

A rearing test was used to assess general motor function [60, 61]. The number of rearing episodes that mice made while exploring a clear plastic cylinder for a 3-min period was recorded.

#### Grid-walking test

To assess motor control deficits, we also used a grid-walking task. A wire mesh grid with 1-in. spaces was used to measure the number of incorrect foot placements, i.e., the number of times the foot slipped through the grid [13]. The ability to traverse the wire grid was evaluated at 35, 36, and 37 days after injury. There were three trials daily with a total of nine trials for the test.

#### Adhesive removal test

To assess sensorimotor deficits, we used the adhesive removal test [62]. Briefly, a small rectangular sticker (3 mm × 4 mm) was placed on the front paw, and the time to remove the sticker was measured.

#### Forelimb contraflexion test

The forelimb contraflexion test was performed as previously described [57]. Mice were temporarily suspended by tail above a flat surface, and a score was assigned based on the following criteria; 5 = normal response characterized by outstretched forelimb making contact with the flat surface (normal mice tend to extend their front limbs spontaneously), 4 = mouse turns preferentially to one side while suspended, 3 = unilateral turning behavior and contraflexion of contralateral forelimb to an angle of less than 90° degree from the normal outstretched position, 2 = unilateral turning behavior and contraflexion of contralateral forelimb to an angle of greater than 90° degrees from the normal outstretched position, 1 = unilateral turning behavior and contraflexion of contralateral forelimb to an angle of more than 90° degrees from the normal outstretched position with no effort made to use limb to prevent a perceived fall.

### Statistical analysis

Statistical analysis was performed using SPSS v.19 with base, regression, advanced models plug-ins (IBM), and missing values plug-ins. Principal component analysis was performed in SPSS and the R Shiny package for principal component analysis (PCA) syndromic plots. Principal components (PCs) were extracted using eigenvalue decomposition of the correlation matrix. PC1 was retained using established statistical rules of (1) eigenvalue >1, (2) scree plot, (3) PC overdetermination with multiple loadings >|0.6|. For PC interpretation, we considered all loadings >|0.3|. PC scores were calculated using the regression method, and hypothesized group difference used GraphPad Prism 5 was used for bar graphs (GraphPad Software, La Jolla, CA). Data are expressed as means ± SEM. Two-way ANOVA was used for flow cytometric analysis followed by Tukey’s post hoc test. One-way ANOVA was used for in vitro cell culture experiment and histological analysis followed by Tukey’s post hoc test. Two-group comparisons were done by unpaired *T* tests. Statistical significance was defined at *p* ≤ 0.05. Statistical results are presented in the figure legends.

## Results

### EVT901 treatment reduces the absolute number of p75NTR-expressing leukocytes in the circulation after TBI in mouse

**Fig. 1:**
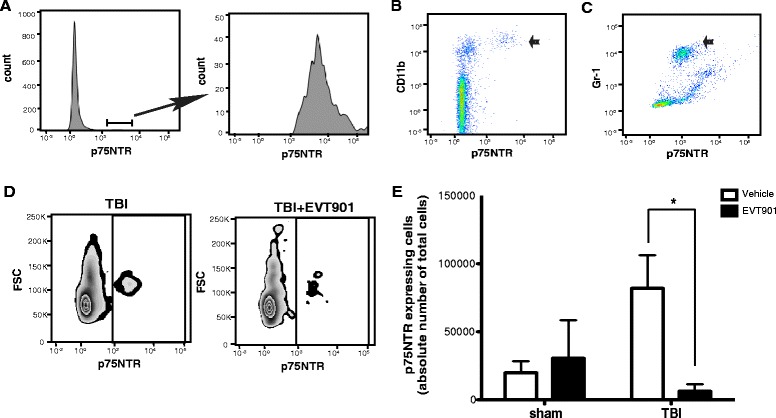
EVT901 treatment reduced p75NTR-expressing leukocyte in the circulation at 7 days after TBI. **a**–**c** Leukocytes were obtained from normal C57Bl/6 mouse blood, and cell type was identified by flow cytometry. p75NTR is expressed on CD11b+ cells (**b**) and Gr-1+ cells (**c**). **d**, **e** Blood was obtained from C57Bl/6 mice at 7 days after TBI with or without EVT901 treatment and isolated leukocytes were immuno-stained with p75NTR Ab. TBI increased the absolute number of p75NTR+ cells, while EVT901 treatment significantly reduced the number in the circulation. One-way ANOVA showed a significant effect of condition (*F*_3,9_ = 4.62, *p* = 0.03, *η* ^2^ = 0.61, power = 0.71; Tukey’s post hoc test: TBI vehicle vs TBI-EVT903, *p* = 0.03). *n* = 2–3 for sham and *n* = 4 for TBI. *Asterisk* indicates *p* < 0.05

Several studies have reported that human leukocytes express p75NTR [44, 45], raising the possibility that p75NTR may be involved in immune function and that EVT901 may have peripheral effects on inflammation after TBI. Mouse leukocytes express p75NTR (Fig. 1). Both CD11b+ and Gr1+ populations isolated using flow cytometry included p75NTR+ cells, and the number of p75NTR+ cells was increased at 7 days post-TBI (Fig. 1). Treatment for 7 days with EVT901 blocked this increase. These data support the possibility that p75NTR could be involved in mediating the early inflammatory responses in the circulation after TBI.

### Blocking p75NTR reduces the pro-inflammatory response of monocytes to LPS in vitro

**Fig. 2:**
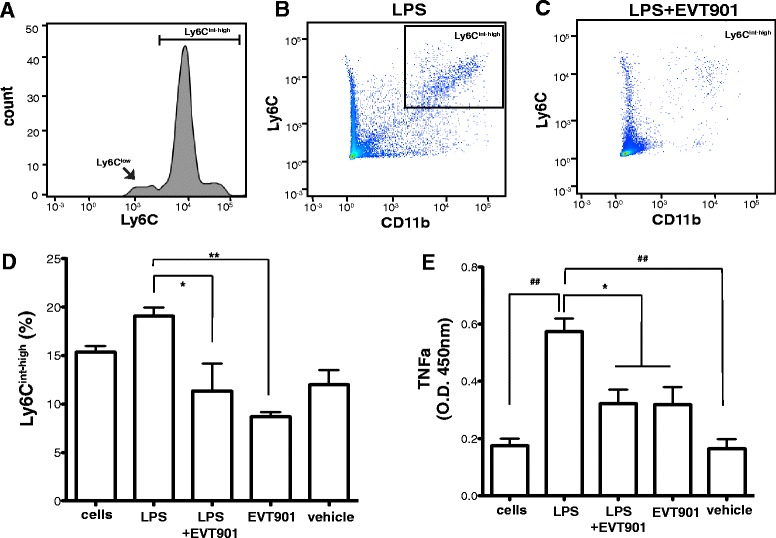
EVT901 treatment inhibited the inflammatory response to LPS in isolated leukocytes in vitro. The isolated leukocytes (1 × 10^6^ cells) were treated with 10 ng/ml LPS, LPS + EVT901 (30 nM), EVT901, or vehicle. After 24-h incubation at 37 °C, cells were isolated by centrifugation and the cell type identified by flow cytometry. **a** CD11b + Ly6C+ monocytes consisted of two distinct subpopulations: Ly6C^low^ monocytes and Ly6C^int-high^ pro-inflammatory monocytes. **b**–**d** LPS treatment increased the proportion of the population that was the CD11b + Ly6C^int-high^, while EVT901 treatment reduced it. Data were obtained from three independent experiments. One-way ANOVA showed a significant effect of condition (*F*_4,14_ = 7.03, *p* = 0.007, *η* ^2^ = 0.76, power = 0.92; Tukey’s post hoc test, *p < 0.05, **p < 0.005). **e** TNFα in the supernatant was measured using ELISA. EVT901 treatment inhibited TNFα production by LPS. Data were obtained from three independent experiments. One-way ANOVA showed a significant effect of condition (*F*_4,15_ = 13.81, *p* = 0.0004, *η* ^2^ = 0.85, power = 0.999; Tukey’s post hoc tests,*p < 0.05, ^*##*^ *p* < 0.001)

In order to test whether p75NTR might mediate pro-inflammatory responses in the periphery, we asked whether EVT901 would affect the response of pro-inflammatory monocytes in vitro to LPS stimulation*.* Since Ly6C^high^ and Ly6C^int^ monocytes are characterized as the “pro-inflammatory monocytes” [63–68], we evaluated the population of CD11b + Ly6C^int-high^ inflammatory monocytes in the isolated leukocytes. Treatment with 10 ng/ml of LPS increased the population of CD11b + Ly6C^int-high^ pro-inflammatory monocytes in the isolated leukocytes (Fig. 2b–d). In order to determine if p75NTR is involved in the differentiation of CD11b + Ly6C^int-high^ inflammatory monocyte, cultures were treated with EVT901. Thirty nanometer of EVT901 treatment significantly inhibited the expansion of CD11b + Ly6C^int-high^ inflammatory monocytes by LPS (Fig. 2d).

Elevated TNFα production is a hallmark of pro-inflammatory stimulation [69–71]. We tested whether EVT901 treatment inhibits LPS-induced TNFα production in the isolated leukocytes. LPS increased TNFα production, as determined by TNFα ELISA, and EVT901 treatment significantly reduced TNFα production (Fig. 2e). These results suggest that EVT901 has anti-inflammatory effects through blocking the p75NTR signaling pathway in leukocytes and supports the hypothesis that p75NTR is involved in the pro-inflammatory responses of peripheral monocytes. We next examined the effects of TBI and EVT901 on this population in the circulation in vivo.

### EVT901 treatment inhibits the production of CD11b + Ly6C^int-high^ inflammatory monocytes in the circulation after TBI

**Fig. 3:**
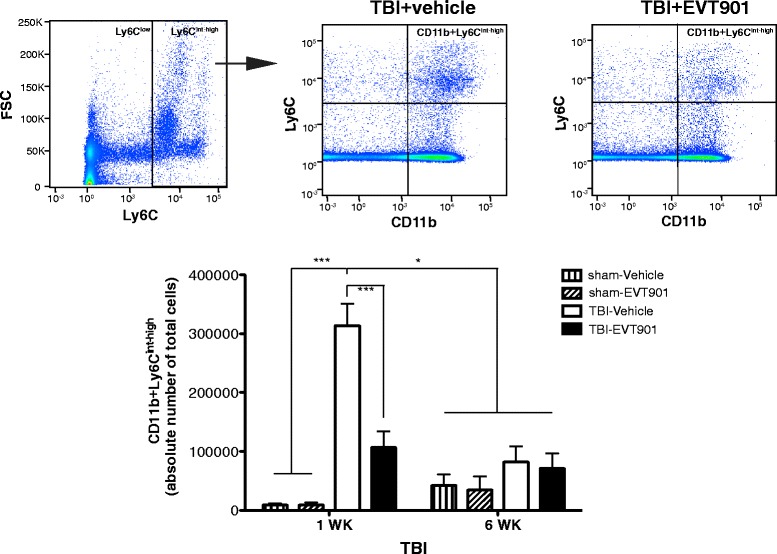
EVT901 treatment reduced the number of Ly6C^int-high^ inflammatory monocytes in the circulation. Blood was obtained from C57Bl/6 WT mice at 1 and 6 weeks after TBI with or without EVT901. Leukocytes were isolated, stained with CD11b Ab and Ly6C Ab, and run on flow cytometry. TBI significantly increased the absolute number of CD11b + Ly6C^int-high^ monocytes in the circulations, while EVT901 treatment reduced the number. Two-way ANOVA revealed a significant effect of condition (*F*_3,25_ = 15.85, *p* < 0.000006, *η* ^2^ = 0.67, power = 1.0), time (*F*_1,32_ = 5.71, *p* = 0.025, *η* ^2^ = 0.19, power = 0.63), time × treatment (*F*_3,32_ = 9.46, *p* < 0.0002, *η* ^2^ = 0.54, power = 0.99). Tukey’s post hoc tests showed there was a significant difference between TBI-vehicle and all other conditions, indicating that TBI increases monocyte numbers at 1 week but not 6 weeks and that EVT901 reduced this effect (*p < 0.05, ***p < 0.001). (*n* = 3 for sham and *n* = 4–7 for TBI)

Ly6C^high^ monocytes have been shown to emerge from the bone marrow and enter the peripheral blood stream where they are directed to sites of tissue injury and inflammation [72, 73]. CCI-TBI dramatically increased the number of circulating CD11b + Ly6C^int-high^ monocytes at 7 days after injury. Further, this effect was significantly reduced by 7-day treatment with EVT901 (Fig. 3). The total absolute number of CD11b + Ly6C^int-high^ inflammatory monocytes was reduced at 6 weeks after TBI, regardless of treatment, suggesting a normalization of pro-inflammatory cell populations in the blood.

### EVT901 treatment reduces the absolute number of total Ly6C^int-high^ monocytes in the injured brain after TBI

**Fig. 4:**
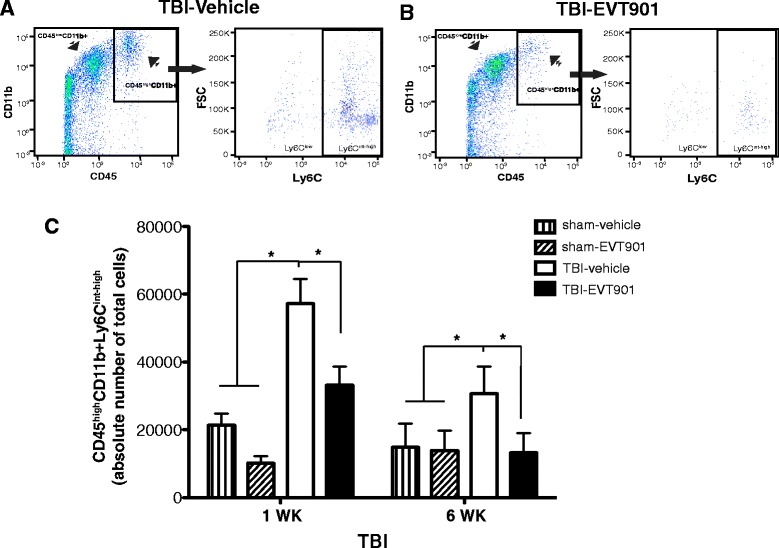
EVT901 treatment reduced the recruitment of Ly6C^int-high^ monocytes into the injured brain at 1 and 6 weeks after TBI. The injured hemisphere was dissected and homogenized. Single cells were obtained using percoll gradients and then stained with Abs against CD45, CD11b+, and Ly6C. **a**–**c** TBI significantly increased the absolute number of Ly6C^int-high^ inflammatory monocytes in the injured brain, while EVT901 treatment reduced the number. Two-way ANOVA revealed significant main effects of treatment (*F*_3,36_ = 6.19, *p* = 0.002, *η* ^2^ = 0.4, power = 0.94) and time (*F*_1,3_ = 5.10, *p* < 0.032, *η* ^2^ = 0.15, power = 0.59). Tukey’s post hoc tests showed that TBI significantly increased CD45^high^CD11b + Ly6C^int-high^ inflammatory monocytes in the injured brain at both 1 and 6 weeks after TBI, while EVT901 treatment reduced the number in the injured brain, *p < 0.05. (*n* = 3–4 for sham and *n* = 5–7 for TBI)

In order to examine if the inhibition of peripheral Ly6C^int-high^ pro-inflammatory monocytes is reflected in a reduction of the recruitment of Ly6C^int-high^ monocytes in the injured brain after TBI, we used flow cytometry to identify those cells from brain homogenates. CD45 Ab was applied along with Abs to CD11b and Ly6C Ab. We were able to distinguish leukocytes of peripheral origin (CD45^high^) and residential microglia (CD45^low^) (Fig. 4a). Here, we analyzed pro-inflammatory monocytes (CD45^high^CD11b + Ly6C^int-high^), which are increased in the injured brain after TBI (Fig. 4a–c). There was a significant reduction of CD45^high^CD11b + Ly6C^int-high^ monocytes in the injured brain after EVT901 treatment compared to vehicle-treated mice after TBI (Fig. 4a–c). These data suggested that p75NTR is involved in not only differentiation of Ly6C^int-high^ pro-inflammatory monocytes in the circulation but also recruitment of these pro-inflammatory monocytes into the injured brain, perhaps by a variety of potential mechanisms. Further, the TBI-induced inflammatory cell increase was maintained at 6 weeks in the brain as opposed to the return to baseline seen in the blood (Fig. 4), and the EVT901 treatment during the first week post-injury reduced that sustained effect in brain as well.

### EVT901 treatment inhibits the recruitment of CCR2+ pro-inflammatory monocytes into the injured brain at 7 days after TBI and is neuroprotective

**Fig. 5:**
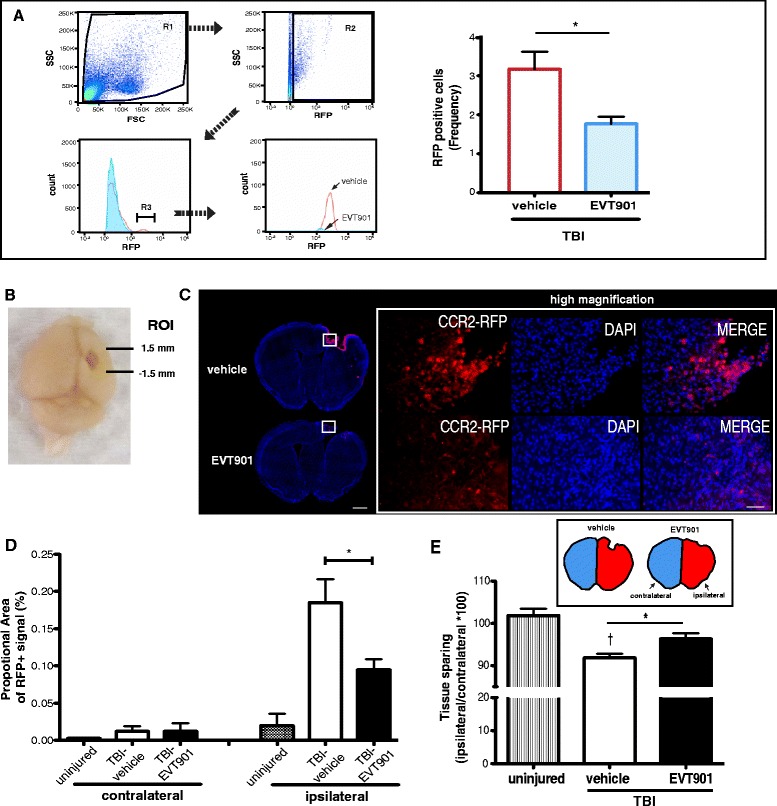
EVT901 treatment reduced CCR2+ monocytes in the injured brain and circulation and reduced tissue damage at 7 days after TBI. **a** Blood was obtained from CCR2^+/RFP^ Tg mice at 7 days after TBI with or without EVT901. One-way ANOVA showed that there was a significant difference between vehicle and EVT901 after injury (*F*_1,8_ = 8.12, *p* = 0.03, *η* ^2^ = 0.58, power = 0.66) (*n* = 3/group). **b** Brain sections from CCR2^+/RFP^ Tg mice were obtained from the lesion area as shown (region of interest (ROI), from 1.5 mm anterior to 1.5 mm posterior of bregma). **c** CCR2^+/RFP^ cells were observed predominantly in the injured area. **d** RFP-positive signal was quantified and the proportional area (P.A. = the area of CCR2-RFP+ signal/total area × 100) is shown. CCR2-RFP-positive signal was highly increased after TBI, and EVT901 treatment significantly reduced it. ANOVA showed a significant effect of side (*F*_1,28_ = 35.03, *p* < 0.001, *η* ^2^ = 0.83, power = 0.999), condition (*F*_2,7_ = 10.97, *p* = 0.007, *η* ^2^ = 0.758, power = 0.922), side × condition (*F*_2,28_ = 7.81, *p* < 0.02, *η* ^2^ = 0.69, *p* = 0.81), and section (*F*_4,28_ = 2.83, *p* < 0.043, *η* ^2^ = 0.288, power = 0.695). **e** EVT901 increased the spared tissue area after TBI. One-way ANOVA showed a significant main effect of condition (*F*_2,7_ = 12.00, *p* = 0.005, *η* ^2^ = 0.774, power = 0.942). Tukey’s post hoc test comparing TBI-vehicle vs TBI-EVT901 was significant (*p = 0.0374), and comparing UN vs TBI-vehicle was also significant (^†^ *p* = 0.005). *Scale bars* in *C* = 1000 μm (*left*) and 100 μm (*right*). (*n* = 4/group)

CCR2, the chemokine receptor for CCL2, is highly expressed by pro-inflammatory monocytes and promotes their recruitment into sites of tissue injury [67, 73]. CCR2 plays a critical role in recruiting Ly6C^high^ monocytes from the bone marrow to the peripheral blood stream, and CCL2 leads monocytes directly to sites of injury and inflammation [72]. Further, a recent study demonstrated that CCR2 signaling is responsible for 80–90 % of the infiltrating monocytes in the injured brain [19]. To evaluate the effects of p75NTR blockade on the recruitment of this specific population of pro-inflammatory monocytes, we used genetically modified CCR2-RFP knock-in mice (CCR2^+/RFP^). The CCR2^+/RFP^ transgenic is a heterozygous mouse with bi-allelic expression of RFP and CCR2 [20, 50]. Because resident microglia do not express CCR2 [20, 50], we were able to use this reporter to identify monocytes of peripheral origin. We were also able to detect RFP-positive cells in the circulation at 7 days after TBI using flow cytometry (Fig. 5a). We found that RFP-positive cells were increased in the circulation at 7 days after TBI and that EVT901 treatment reduced that population (Fig. 5a). RFP-positive cells were also detected in the injured brain at 7 days after TBI (Fig. 5c), as previously reported [20]. To quantify the infiltrating RFP-positive cells in the injured brain, we applied a proportional area (P.A.) measurement based on previous studies [21, 52]. Brain tissue sections were obtained from the region of interest (ROI) around the lesion (Fig. 5b). The RFP-positive signal was highly increased in the injured brain at 7 days after TBI, while EVT901 treatment significantly reduced it (Fig. 5b–d), suggesting that EVT901 treatment inhibits the infiltration of CCR2+ pro-inflammatory monocytes. And consistent with our results in rat [43], we found that treatment with EVT901 for 7 days after TBI increased the amount of spared brain tissue (Fig. 5e). These data suggest that blocking p75NTR with EVT901 reduces the peripheral mobilization and expansion of CCR2+ monocytes after injury, perhaps by reducing the chemokine signaling from the brain by reducing the amount of injury [43]. However, in light of the effects of EVT901 on the induction of pro-inflammatory monocytes in vitro, EVT901 may also act to reduce CCR2+ monocyte expansion more directly by affecting myeloid cell proliferation and differentiation through blocking p75NTR signaling.

**Fig. 6:**
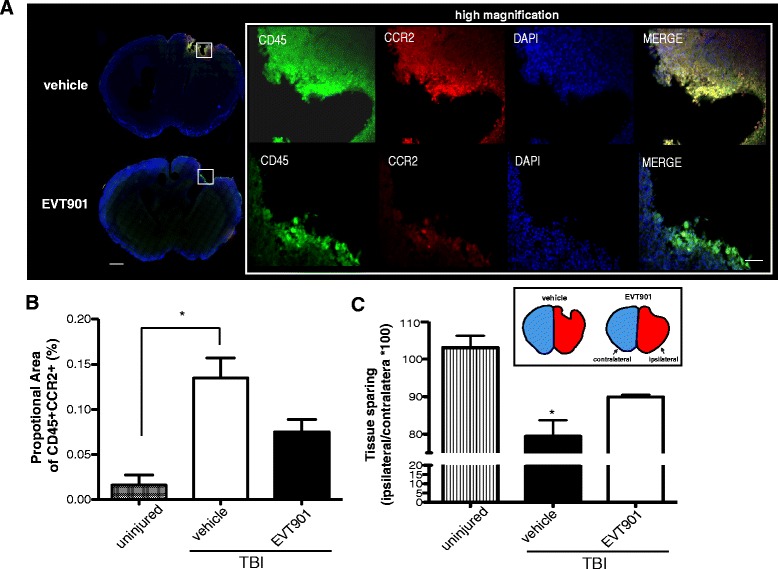
EVT901 treatment reduced CD45 + CCR2+ double-positive monocytes in the injured brain in C57Bl/6 WT mice at 7 days after TBI. **a** Brain tissue sections were immuno-stained with CCR2 Ab and CD45 Ab. **b** TBI increased the CD45 + CCR2+ double-positive signal in the injured area, while EVT901 treatment abrogated this effect. ANOVA revealed a significant main effect of condition (*F*_2,6_ = 14.50, *p* = 0.005, *η* ^2^ = 0.83, power = 0.96) and section (*F*_4,24_ = 5.86, *p* = 0.002, *η* ^2^ = 0.49, power = 0.96). Tukey’s post hoc test showed a significant difference between uninjured and TBI-vehicle (*p* = 0.004). **c** Tissue area was significantly reduced by TBI, and this effect was abrogated by EVT901. One-way ANOVA showed a main effect of condition (*F*_2,6_ = 14.06, *p* = 0.003, *η* ^2^ = 0.82, power = 0.96). Tukey’s post hoc tests showed that TBI-vehilce, but not EVT901, was different from uninjured control (*p* < 0.004). *Scale bar* in *A* = 1000 μm (*left*) and 100 μm (*right*). (*n* = 3 for UN and *n* = 5 for TBI groups)

Because CCR2^+/RFP^ mice are heterozygotes, there may be gene-dose-dependent effects that alter recruitment of macrophages into the tissue [74]. Therefore, we asked if EVT901 has comparable effects in C57BL/6 WT mice using alternative labeling strategies. To identify peripheral monocytes, we used CD45, which is highly expressed on peripheral leukocytes. As an alternative to CCR2^+/RFP^, we used co-localization of CD45 and CCR2 immunostaining. We found that the co-localization of positive signal for CD45 and CCR2 is increased in the injured brain ROI at 7 days after TBI and that EVT901 treatment significantly reduced this signal (Fig. 6a, b). Consistent with CCR2^+/RFP^ Tg mice, tissue sparing (measured as the spared tissue area from the lesion ROI sections) was enhanced in C57Bl/6 WT treated with EVT901 compared to vehicle-treated mice (Fig. 6c). Thus, we were able to confirm the effects of EVT901 on CCR2+ cell recruitment using both Tg and wild-type mice.

### EVT901 treatment reduces F4/80+ monocytes/macrophages in the injured brain after TBI

**Fig. 7:**
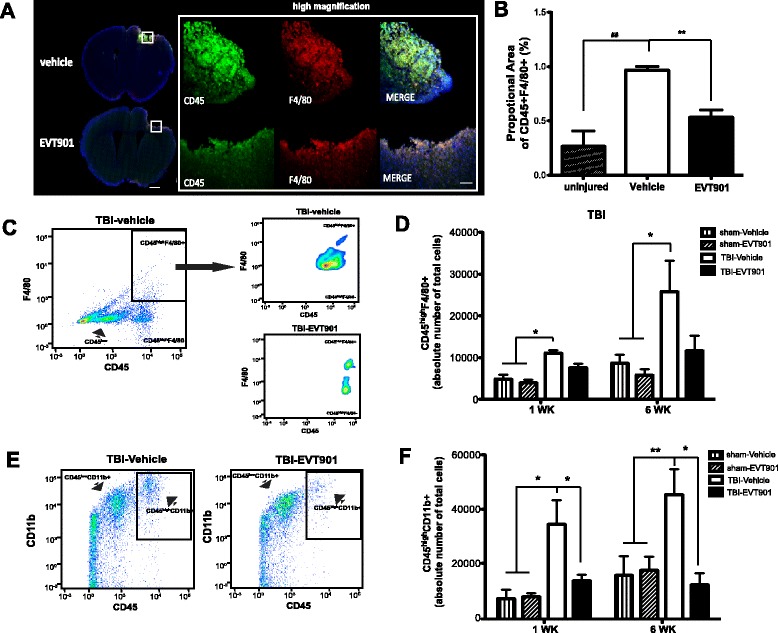
EVT901 treatment attenuated the infiltration of peripheral monocytes/macrophages into the injured brain in WT mice. **a** Brain tissue sections from C57BL/6 mice at 7 days after TBI were immuno-stained with CD45 and F4/80 Abs. **b** TBI increased the proportional area of CD45 + F4/80+ double-positive signal in the injured brain, while EVT901 treatment reduced it. ANOVA indicated that there was a significant treatment effect (*F*_1,6_ = 14.89, *p* = 0.005, *η* ^2^ = 0.83, power = 0.97). Tukey’s post hoc test revealed a significant difference between uninjured vs TBI-vehicle (^##^ *p* = 0.004) and TBI-vehicle vs TBI-EVT901 (**p = 0.006). **c**–**f** Cells isolated from C57Bl/6 WT mice brains at 1 and 6 weeks after sham or TBI, with or without EVT901, were stained with Abs against CD45, CD11b+, and F4/80. **c**, **d** TBI increased the absolute number of CD45^high^F4/80+ monocytes, and EVT901 significantly reduced the number. Two-way ANOVA followed by Tukey’s post hoc tests showed that there was a significant effect of condition (*F*_3,36_ = 4.06, *p* = 0.016, *η* ^2^ = 0.31, power = 0.779), but no effects of time. TBI increased CD45^high^F4/80+ cells in the injured brain compared to sham, while EVT901 treatment abrogated this effect (*p < 0.05). **e**, **f** TBI significantly increased the absolute number of CD45^high^CD11b+ monocytes in the injured brain, and EVT901 significantly reduced it. Two-way ANOVA showed a significant effect of condition (*F*_3,35_ = 9.02, *p* = 0.0002, *η* ^2^ = 0.5, power = 0.99), but no effect of time. Tukey’s post hoc tests showed a significant effect of injury and EVT901 at both 1 and 6 weeks (*p < 0.05, **p < 0.001 compared to TBI-vehicle). *Scale bar* in *A* = 1000 (*left*) and 100 μm (*right*). (*n* = 3–4 for sham and *n* = 5–7 for TBI)

A recent study shows that CCR2 is required for recruiting monocytes from the peripheral circulation into the brain in the first few days after TBI [19]. However, since CCR2 is rapidly down-regulated when CCR2 monocytes differentiate into tissue resident macrophages, it is only possible to detect newly arrived CCR2 monocytes in the tissues [20, 50]. To overcome this issue, F4/80, a marker for mature monocytes/macrophages, was used to estimate the total population of infiltrating peripheral monocytes in the injured brain at 7 days after TBI. We found that the double-positive signal of CD45 and F4/80 was increased in the injured brain at 7 days after TBI (Fig. 7a) and that EVT901 treatment significantly reduced this signal (Fig. 7a, b).

To confirm the immunohistochemical analysis, we applied flow cytometric analysis of brain homogenates, which allowed us to estimate the absolute total number of infiltrating peripheral monocytes/macrophages in the injured brain. We found that the absolute total number of CD45^high^F4/80+ cells was increased in the injured brain at 1 and 6 weeks after TBI, and yet again, EVT901 treatment significantly reduced these populations (Fig. 7c, d). In addition, we examined total monocyte/macrophage populations in the injured brain using CD11b Ab, a common marker for both immature and mature monocytes and macrophages. We found that there was a significant increase in CD45^high^CD11b+ monocytes in the injured brain (Fig. 7e, f), and this increase, present at both 1 and 6 weeks, was abrogated by the 1-week treatment with EVT901. In contrast, the total number of CD45^low^CD11b+ cells, which represent the resident microglia, was not affected by TBI or EVT901 (data not shown).

### Blocking p75NTR signaling by EVT901 treatment improves motor function after TBI and modifies the multivariate syndromic effects of TBI on both immune system and behavior

Previous studies show that prevention of early trafficking of peripheral immune cells into the injured CNS results in better behavioral outcomes and reduction of tissue damage [13, 19, 20, 22–24].

**Fig. 8:**
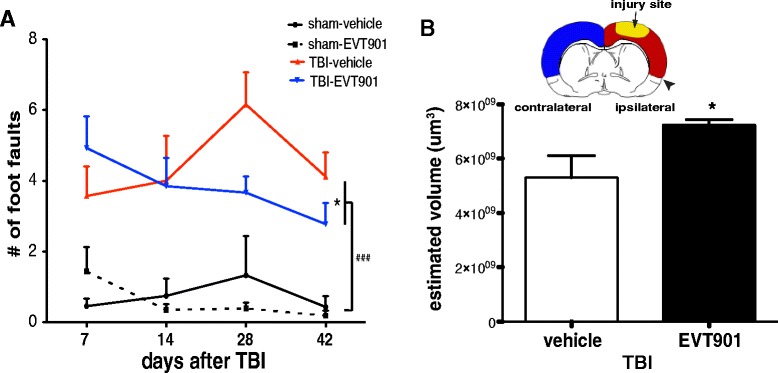
EVT901 treatment improved motor function and reduced brain damage after TBI. **a** Persistent fine motor coordination was evaluated by beam walking for 6 weeks after TBI. The total number of footfaults was increased by TBI, and EVT901 reduced this deficit. Two-way ANOVA revealed a significant main effect of injury (*F*_1,26_ = 48.996, *p* = 0.0000002, *η* ^2^ = 0.65, power = 1), and a significant time × treatment effect (*F*_3,78_ = 4.90, *p* = 0.04, *η* ^2^ = 0.16, power = 0.9). Tukey’s post hoc tests revealed a significant difference between TBI-vehicle and TBI-EVT901 (*p = 0.04) and sham and TBI (^###^ *p* < 0.0001). **b** Unbiased stereological volumetric analysis showed a significant atrophy of cortex on the ipsilateral, but not contralateral side of TBI. EVT901 treatment inhibited tissue atrophy compared to vehicle. *T* test (^*^ *p* = 0.04). (*n* = 5 per group)

The CCI-TBI protocol we used in the mouse in the present study resulted in relatively minor motor deficits that largely recovered over the 6 weeks after injury. However, we did see evidence for a drug effect on some measures. To analyze the full set of behavioral and associated inflammatory biomarkers, we performed principal components analysis (PCA) to extract the global syndromic outcome patterns. Consistent with these prior studies, we found that blocking early trafficking of peripheral monocytes into the CNS by EVT901 treatment after TBI reduced neurological deficits. Using a battery of behavioral tests with TBI mice, we found that fine motor functions performed on a beam were significantly improved by EVT901 administered for 1 week after injury (Fig. 8c, d). Further, we found that TBI significantly reduced the total volume of neocortex at 6 weeks, and EVT901 significantly reversed this when compared to the vehicle-treated brain (Fig. 8b).

**Fig. 9:**
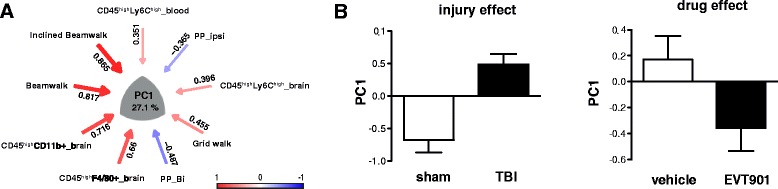
Multivariate principal component analysis (PCA). To understand the relationship of behavioral outcome and innate immune cell responses at 42 days after TBI, PCA was used. This analysis yielded five dimensional PC-loading patterns accounting for 80.55 % of total variance (details in Additional file 1: Figure S1). **a** Significant loading on PC1 indicated that more errors on the beam-walking tasks were associated with the numbers of peripheral inflammatory immune cells in the brain and (less so) with increased pro-inflammatory monocytes in the blood. Bilateral paw placement, a measure of recovery of cortical function, was inversely correlated between individual variables and PC1. *Red arrow* indicates a positive relationship, while *blue arrow* indicates negative relationship. PCs 2–5 also showed associations between behavioral recoveries and inflammatory markers (see Additional file 1: Figure S1). **b** Post hoc analysis of the main effects on PC1 (27.1 % of total variance) indicated that there is a significant effect of injury (*F*_1,39_ = 21.412, *p* = 0.000, *η* ^2^ = 0.380, power = 0.994) and of EVT901 treatment (*F*_1,39_ = 4.435, *p* = 0.042, *η* ^2^ = 0.112, power = 0.535)

The PCA revealed five orthogonal principal components that partitioned the shared variance between cellular and behavioral outcome measures at 6 weeks (Fig. 9, and Additional file 1: Figure S1). PC1, which accounted for 27 % of the total variance, appeared to describe the coordinate increase in inflammatory markers and the deterioration of behavioral function seen after TBI (Fig. 9a), providing a useful composite metric for the syndrome of CCI-TBI in our mice: comparisons of sham and TBI subjects using PC1 as a univariate measure showed a significant increase in value (Fig. 9b). One week of treatment with EVT901 dramatically lowered the PC1 z-score values in mice with TBI, supporting the idea that EVT9011 coordinately affected neuroinflammation and behavioral outcomes.

## Discussion

Our previous study using the novel p75NTR antagonist EVT901 was initially aimed at blocking the well-documented effects of p75NTR signaling on apoptotic cell death in CNS neurons and glia, and we showed quite dramatic effects on measures of tissue sparing and a variety of neurological outcomes in multiple models of TBI in the rat [43]. The results also showed very dramatic reductions in histological measures of neuroinflammation and microglial activation. We wondered whether EVT901 might be affecting neuroinflammatory signals as well as apoptosis. However, we saw no evidence for microglial expression of p75NTR in that study, and aside from a few reports of p75NTR on microglial cells in vitro [75, 76], there is little evidence for microglial expression of p75NTR in the adult brain. However, there have been isolated reports of p75NTR expression in leukocytes [45, 46], (reviewed in [44]). We thus raised the question as to whether p75NTR could also be involved in the peripheral inflammation seen after TBI, with consequences for the recruitment of monocytes/macrophages into the injured brain. We found in mouse TBI, a strong pro-inflammatory peripheral acute response to CNS injury, and the accumulation of peripheral leukocytes, including pro-inflammatory monocytes and CCR2+ cells, in the brain injury site at 1 and 6 weeks after injury.

Seven days of treatment with EVT901 reduced the effects of each element of the combined blood and brain inflammatory response to CCI-TBI: it reduced the increased expression of p75NTR on peripheral monocytes, it reduced the increase in numbers of pro-inflammatory monocytes in the blood at 1 week after injury, and it reduced the invasion of peripheral myeloid cells (defined by multiple phenotypic markers) into the brain. And consistent with our previous work [43], EVT901 was neuroprotective and reduced post-TBI neurological deficits.

These results are consistent with the hypothesis that EVT901 has both central and peripheral effects on inflammation by blocking p75NTR signaling in both neural and peripheral immune cells, dampening the effects of central neuroinflammation by reducing both the CNS damage caused by p75NTR apoptotic signaling and the exacerbation of initial microglial activation by the production and invasion of highly pro-inflammatory peripheral monocytes. While the reduction in peripheral inflammatory responses might be in part an indirect effect of the reduction of central injury, and therefore of signaling (e.g., by DAMPs, CCL2, cytokines, or other mediators), the strong inhibition by EVT901 of pro-inflammatory responses to LPS in isolated monocytes in vitro provides evidence for a direct effect.

### The choice of injury model

We chose to use a controlled-cortical impact (CCI) model of TBI in the mouse for these studies. These injuries are characterized by frank contusions of the cortical mantle and disruption of the local blood-brain barrier, although widespread diffuse injury also has been documented [51, 77]. A significant proportion of human TBI includes focal contusion injuries that may “bloom” and expand over the first few days after injury [78, 79]. These injuries, with their frank breach of the blood-brain barrier, are likely to be especially affected by invasion of peripheral myeloid cells. Further, the presence of an MRI-defined contusion injury in mild TBI is a strong predictor of negative outcome [80]. Thus, treatments that reduce the evolution of contusion injuries are likely to be translatable to at least an important and potentially treatable subset of human TBI.

### Leukocyte invasion in TBI

Accumulating evidence shows that infiltration of peripheral monocytes/macrophages as well as neutrophils is an important part of the inflammatory response to TBI. Massive infiltration of peripheral immune cells into the injured brain after TBI is a prevalent outcome resulting in significant promotion of cell death and tissue damage, inhibition of the wound healing process, and impairment of neurological recovery. And several studies show that blocking trafficking of peripheral monocytes into the injured CNS results in better behavioral outcomes and reduced tissue damage [13, 19, 20, 22, 24, 49, 81, 82]. The data from our prior work [13] and the current set of studies in the mouse supports this view and also suggests that the proliferation and differentiation of pro-inflammatory monocytes in the circulation in response to TBI contributes to the availability of pro-inflammatory cells for recruitment to the brain. Somewhat in contrast to these data, a recent study reports that a closed head injury TBI did not significantly increase the population of blood monocytes and saw a reduction in F4/80 macrophages at 30 days post-TBI. They also noted increases in the anti-inflammatory M2 monocytes populations in the circulation, suggesting that TBI produces an immune-suppressive environment by reducing blood monocytes as well as thymocytes [83]. That study used several monocyte markers (B220^−^CD4^−^CD8^−^Nk1.1^−^Ly6G^−^CD11b^+^CD115^+^) to identify the monocyte population, while we used CD11b + Ly6C^high^ for inflammatory monocytes, which are increased at 1 week, but not 6 weeks, after TBI in the circulation (Fig. 3). In addition, we also found that TBI increases the population CD11b + F4/80+ mature monocytes in the circulation at 1 week (unpublished data). The apparent discrepancy may be due to the different markers used to identify different subsets of peripheral monocytes, as peripheral monocytes generally display heterogeneous cell markers on their surface depending on their stage of maturity (i.e., immature vs mature), activation state (activated vs non-activated), and polarization (pro-inflammatory M1 monocytes vs anti-inflammatory M2 monocytes). In the current study, we only investigated the pro-inflammatory monocyte populations as identified by CD11b + Ly6C^int-high^ after TBI. It is also likely that the initial pro-inflammatory response we noted leads to a more chronic, immune-suppressive phenotype as also noted in chronic SCI [25]. However, it is also clear that there is a wide range of phenotypes that evolve from the peripheral monocyte invasion of injured CNS (e.g., [49], and some of these are clearly neuroprotective and promote repair [82].

While it is known that TBI and other CNS injuries also produce an acute and persistent peripheral inflammatory response, the role of the peripheral immune system in exacerbating or ameliorating the CNS injury is not well understood [14], although much evidence suggests that peripheral inflammation can influence CNS function even in the absence of injury [84, 85]. The results of the current studies are consistent with the idea that CNS injury produces signals that stimulate the expansion of bone marrow myeloid cells via CCL2 signaling via CCR2 [72, 73], DAMPs [14], or other mediators.

### p75NTR and inflammation

The mechanisms of p75NTR signaling in inflammation are not known, and the possibility of p75NTR as an inflammatory mediator has barely been explored [44–46]. In the present study, blocking p75NTR with EVT901 reduced the production of TNF-α in response to LPS. EVT901 also reduced the LPS and pro-NGF increase in p75NTR expression. We previously showed that EVT901 also reduces the increase in p75NTR expression induced by pro-NGF stimulation in cultured oligodendrocytes and oligodendrocyte precursor cells [43], which accompanied (and perhaps mediated) the reduced apoptotic cell death produced by pro-NGF in these cells. The mechanism for the autocrine-like response is unknown. Further, we do not know if the p75NTR signaling pathway in myeloid cells leads to apoptosis in peripheral monocytes as it does in neurons an oligodendrocytes [32, 86]. However, Choi and Freidman [87, 88] have shown that the inflammatory cytokines IL-1β and TNF-α increase p75NTR expression in neurons and astrocytes. This study emphasized that IL-1β induces p75NTR through the p38MAPK pathway, while TNF-α activates the NF-κB pathway in neurons and astrocytes, promoting neuronal cell death and glia proliferation, respectively. The production of high levels of IL-1β and TNF-α in both the CNS and periphery after TBI thus may be involved in the rather pronounced increases in p75NTR expression in monocytes that we see after TBI. LPS is recognized by monocytes and macrophages through toll-like receptor (TLRs), which are involved in the innate immune response to LPS by producing inflammatory cytokines, such as TNF-α [89]. LPS has been shown to induce p75NTR expression on monocyte-derived dendritic cells through the NF-κB signaling pathway, suggesting that p75NTR expression is enhanced by DAMPs or other pro-inflammatory stimuli [90]. Both TNF-α and IL-1β receptors are present on monocytes [91, 92], and IL-1β and TNF are associated with NF-κB signaling in monocytes as well [89, 93, 94]. We have not yet tested these various signaling pathways with respect to the effects of EVT901 on peripheral monocytes.

While the evidence for a peripheral role of p75NTR in peripheral inflammation is growing, the principal evidence in the present study comes from the modulation of immune functions by EVT901. Since p75NTR is a member of the TNF receptor superfamily (TNFRSF 16) [95], the potential for off-target effects, TNFR1 or 2 for example, should be considered. However, extensive analysis of EVT901 binding and effects on a variety of cognate and mutated TNFRSF constructs in vitro showed a high degree of specificity of EVT901 for p75NTR [43]. Nevertheless, further studies using converging mechanisms to block p75NTR signaling are certainly warranted. Further, the ligand(s) involved in peripheral immune actions of p75NTR are not known. In the CNS, pro-NGF, pro-BDNF, beta-amyloid, and PrP have been shown to bind and activate p75NTR signaling, and this depends on the co-receptors involved including tropomyosin receptor kinases (Trks) and sortilin [30, 32]. These same mediators may be present in the periphery, and there is the possibility that the release from sites of CNS injury could play a role in the primary activation of peripheral monocytes, but this remains to be determined. Other possibilities for the role of p75NTR might involve proteolytic processing and subsequent intracellular signaling (e.g., see [96]. This is an intriguing possibility in light of the apparent interaction between TNF and p75NTR, since TACE and MMPs are involved in proteolytic cleavage-related modulation of signaling in both systems [33, 97, 98]. Another possibility is that p75NTR can be involved in cell migration and proliferation by interacting with α9β1 integrin [99], suggesting that it may be contributing to recruitment of peripheral immune cells into the CNS tissues. However, the role of α9β1 integrin has yet to be described after CNS injury.

In addition, there is no evidence of p75NTR expression on microglia in the adult normal or injured CNS. We have found that EVT901 did not affect the number of CD45^low^CD11b+ or F4/80+ microglia in the injured brain by flow cytometric analysis. Further, our attempt to detect p75NTR expression in microglia in the injured brain failed. Thus, we have reached the conclusion that the effect of EVT901 is not directly on microglia in the injured brain but rather on the peripheral immune cells. Previously, we have seen that EVT901 treatment reduced microglial activation in the injured rat brain after TBI and conclude that this effect is probably due to EVT901 reducing neuronal and oligodendrocyte cell death, as well as reducing trafficking of pro-inflammatory peripheral monocytes into the injured brain.

## Conclusions

We show that attenuation of peripheral Ly6C^int-high^ pro-inflammatory monocytes by EVT901 treatment is associated with a reduction in trafficking of Ly6C^int-high^ inflammatory monocytes into the injured brain and subsequent improvement of motor function (Figs. 3, 4, 5, 6, 7, and 8). There is strong evidence that EVT901 targets p75NTR dimerization and signaling in in vitro studies and in vivo in the CNS [43]. While further studies will be needed to confirm that p75NTR is the primary target of EVT901 in immune cells, the data in this study in combination with the rather limited literature on the role of p75NTR in immune cells raises the possibility that p75NTR is an important mediator of peripheral inflammation as well as a diverse, co-receptor-dependent modulator of neuronal survival and growth in the CNS. Beyond a recent report of co-localization of p75NTR and trkA in human monocytes/macrophages [44], little is known about the presence of p75NTR co-effectors in immune cells, and the role(s) of p75NTR in peripheral immunity may be as diverse as in the CNS. However, the strong modulatory effects of EVT901 on both central and peripheral inflammation suggest that p75NTR represents a novel target for immune modulation that favors neuroprotection and recovery after CNS injury.

## Additional file

Additional file 1: Figure S1. Complete PCA solution. To understand the relationship of behavioral outcome and innate immune cell responses, multivariate PC analysis was used. (A) PCA applies eigenvalue decomposition to the entire matrix cross correlations of all outcomes and clusters variables that move together as an integrated, optimally weighted linear composite (PC) to maximally explain the variance in cross-correlation. Once the first eigenvalue decomposition (PC1) is extracted, a second orthogonal (uncorrelated) decomposition calculated accounting for the second highest cluster of variance (PC2) and so on until all of the variance in the data set is partitioned into sequentially numbered PCs. The PC measures are rank ordered by the proportion of the total variance in the dataset that they explain. Standardized statistical PC retention rules are then applied to retain ‘significant’ PCs with eigenvalues > 1 and (B) PCs above the ‘elbow of the scree plot. (C) Interpretation of PC content based on *PC loadings*, which are equivalent to a pearson correlation between each variable and the PC composites. The PC loading pattern shows the contribution every single variable (outcome measures) on the extracted PCs. This analysis yielded 5 dimensional PC loading patterns (total, 80.55 % of variance). The PC1 module represented the relationship between neurological outcome and pro-inflammatory monocyte responses (27.13 % of variance). The individual *PC score* (see Fig. 9b) for each subject is the sum of the multiplied loading by the individual raw value of single outcome measures. The individual PC scores are used to define the impact of experimental manipulations on the relationship between outcome measures. (PDF 91 kb)

## Abbreviations

AD: Alzheimer’s disease
BDNF: brain-derived neurotrophic factor
IL-1β: interleukin-1 beta
IL-6: interleukin-6
MAG: myelin-associated glycoprotein
NGF: nerve growth factor
NT: neurotrophin
OMgp: oligodendrocyte myelin glycoprotein
p75NTR: p75 neurotrophin receptor
PBMC: peripheral blood mononuclear cell
TBI: traumatic brain injury
TNF-α: tumor necrosis factor-alpha
Trk A, B, C: tropomyosin receptor kinase A, B, C
