# Supplementary figures and images for: A novel antagonist of p75NTR reduces peripheral expansion and CNS trafficking of pro-inflammatory monocytes and spares function after traumatic brain injury
Source: J Neuroinflammation. 2016 Apr 22;13:88. doi: 10.1186/s12974-016-0544-4 (PMC4840857; doi:10.1186/s12974-016-0544-4)

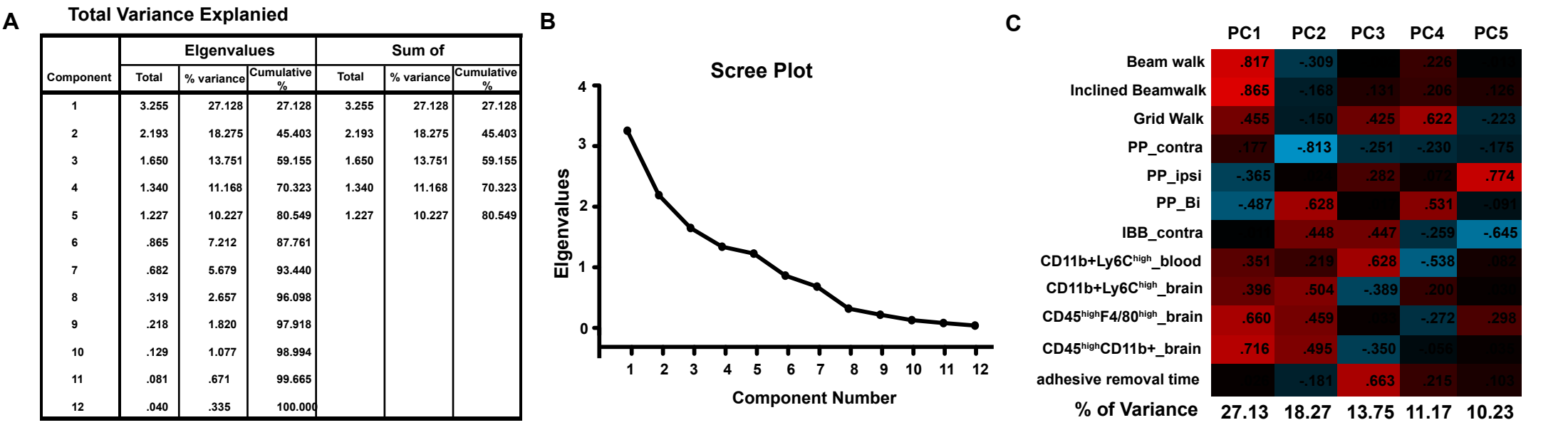

Lee et al., Supplemental Figure. 1

Supplement: Additional file 1: Figure S1. — Complete PCA solution. To understand the relationship of behavioral outcome and innate immune cell responses, multivariate PC analysis was used. (A) PCA applies eigenvalue decomposition to the entire matrix cross correlations of all outcomes and clusters variables that move together as an integrated, optimally weighted linear composite (PC) to maximally explain the variance in cross-correlation. Once the first eigenvalue decomposition (PC1) is extracted, a second orthogonal (uncorrelated) decomposition calculated accounting for the second highest cluster of variance (PC2) and so on until all of the variance in the data set is partitioned into sequentially numbered PCs. The PC measures are rank ordered by the proportion of the total variance in the dataset that they explain. Standardized statistical PC retention rules are then applied to retain ‘significant’ PCs with eigenvalues > 1 and (B) PCs above the ‘elbow of the scree plot. (C) Interpretation of PC content based on PC loadings, which are equivalent to a pearson correlation between each variable and the PC composites. The PC loading pattern shows the contribution every single variable (outcome measures) on the extracted PCs. This analysis yielded 5 dimensional PC loading patterns (total, 80.55 % of variance). The PC1 module represented the relationship between neurological outcome and pro-inflammatory monocyte responses (27.13 % of variance). The individual PC score (see Fig. 9b) for each subject is the sum of the multiplied loading by the individual raw value of single outcome measures. The individual PC scores are used to define the impact of experimental manipulations on the relationship between outcome measures. (PDF 91 kb) [file 12974_2016_544_MOESM1_ESM.pdf]
